# Supplementary material for: MetaRibo-Seq measures translation in microbiomes
Source: Nat Commun. 2020 Jun 29;11:3268. doi: 10.1038/s41467-020-17081-z (PMC7324362; doi:10.1038/s41467-020-17081-z)
Supplement: Supplementary file 10 — Supplementary Data 7 [file 41467_2020_17081_MOESM10_ESM.zip › File2/Confidence_VeryHigh_Taxonomy/367706_out.krona.html]

Javascript must be enabled to view this page.

members
magnitude
magnitudeUnassigned
count
unassigned
taxon
rank

367706\_out

50

50
2
superkingdom

50
1239
phylum

909932
1
class

order
1
1843489

1
31977
family

1
39948
genus

species

SRS063985\_contig\_number\_16591
1
1955814

186801
48
class

186802
48
order

family
541000
2

species

SRS075078\_contig\_number\_13482
1
2292270

genus
1
216851

species

SRS075821\_contig\_number\_16902
1
853

1
19
186803
family

SRS016495\_contig\_number\_10460

17
1898203
species

SRS011239\_contig\_number\_16593SRS012849\_contig\_number\_29102SRS013476\_contig\_number\_33063SRS015431\_contig\_number\_37315SRS015890\_contig\_number\_19187SRS019496\_contig\_number\_17875SRS023583\_contig\_number\_contig-100\_199.101387SRS050026\_contig\_number\_9142SRS050998\_contig\_number\_11529SRS058070\_contig\_number\_23688SRS078419\_contig\_number\_12766SRS104311\_contig\_number\_10486SRS1054716\_contig\_number\_6274SRS144362\_contig\_number\_35615SRS147346\_contig\_number\_64905SRS147614\_contig\_number\_541SRS148319\_contig\_number\_contig-100\_2383.89410

genus

SRS144362\_contig\_number\_30604
1
841

family
31979
27

genus

SRS053398\_contig\_number\_25727
27
1
1485


SRS014459\_contig\_number\_17559SRS015854\_contig\_number\_16728SRS019445\_contig\_number\_17523SRS050752\_contig\_number\_31554SRS063040\_contig\_number\_37022SRS077194\_contig\_number\_23195SRS077641\_contig\_number\_22918SRS078419\_contig\_number\_28624SRS1041038\_contig\_number\_17644SRS143342\_contig\_number\_2847SRS143372\_contig\_number\_8518SRS146764\_contig\_number\_32831SRS893230\_contig\_number\_11015SRS893342\_contig\_number\_4634SRS893366\_contig\_number\_20277SRS893373\_contig\_number\_22306SRS971276\_contig\_number\_30574
species
59620
17


SRS143876\_contig\_number\_7678
species
1262805
1

3
1262845
species

SRS019068\_contig\_number\_60684SRS019693\_contig\_number\_9312SRS1041136\_contig\_number\_24916

2293014
4

SRS022609\_contig\_number\_47070SRS056273\_contig\_number\_contig-100\_77.203437SRS097920\_contig\_number\_contig-100\_1464.182262SRS098827\_contig\_number\_6400
species


SRS047014\_contig\_number\_31938
species
2293030
1


SRS020869\_contig\_number\_3681
species
1897036
1
